# Ulcerogenic *Helicobacter pylori* Strains Isolated from Children: A Contribution to Get Insight into the Virulence of the Bacteria

**DOI:** 10.1371/journal.pone.0026265

**Published:** 2011-10-19

**Authors:** Inês Vitoriano, Kathy D. Saraiva-Pava, Alexandra Rocha-Gonçalves, Andrea Santos, Ana I. Lopes, Mónica Oleastro, Mónica Roxo-Rosa

**Affiliations:** 1 Faculdade de Engenharia, Universidade Católica Portuguesa, Rio de Mouro, Portugal; 2 Chymiotechnon, Departamento de Química, Universidade de Coimbra, Coimbra, Portugal; 3 Departamento de Doenças Infecciosas, Instituto Nacional Saúde Dr. Ricardo Jorge, Lisboa, Portugal; 4 Departamento de Pediatria, Hospital Universitário de Santa Maria/Faculdade de Medicina de Lisboa, Lisboa, Portugal; Veterans Affairs Medical Center (111D), United States of America

## Abstract

Infection with *Helicobacter pylori* is the major cause for the development of peptic ulcer disease (PUD). In children, with no other etiology for the disease, this rare event occurs shortly after infection. In these young patients, habits of smoking, diet, consumption of alcohol and non-steroid anti-inflammatory drugs and stress, in addition to the genetic susceptibility of the patient, represent a minor influence. Accordingly, the virulence of the implicated *H. pylori* strain should play a crucial role in the development of PUD. Corroborating this, our *in vitro* infection assays comparing a pool of five *H. pylori* strains isolated from children with PUD to a pool of five other pediatric clinical isolates associated with non-ulcer dyspepsia (NUD) showed the greater ability of PUD strains to induce a marked decrease in the viability of gastric cells and to cause severe damage in the cells cytoskeleton as well as an impairment in the production/secretion of mucins. To uncover virulence features, we compared the proteome of these two groups of *H. pylori* strains. Two-dimensional gel electrophoresis followed by mass-spectrometry allowed us to detect 27 differentially expressed proteins between them. In addition to the presence of genes encoding well established virulence factors, namely *cagA*, *vacA*s1, *oipA* “on” status, *homB* and *jhp562* genes, the pediatric ulcerogenic strains shared a proteome profile characterized by changes in the abundance of: motility-associated proteins, accounting for higher motility; antioxidant proteins, which may confer increased resistance to inflammation; and enzymes involved in key steps in the metabolism of glucose, amino acids and urea, which may be advantageous to face fluctuations of nutrients. In conclusion, the enhanced virulence of the pediatric ulcerogenic *H. pylori* strains may result from a synergy between their natural ability to better adapt to the hostile human stomach and the expression of the established virulence factors.

## Introduction

*Helicobacter pylori* is a spiral-shaped, microaerophilic, gram-negative bacterium that inhabits the human stomach. Depending on the socioeconomic status of the country, the prevalence of infection varies from 40 to over 80% of the population, with higher rates for developing countries [1]. Infection is usually acquired during childhood and always elicits an acute immune response which, in the absence of effective treatment, persists throughout the patient's life, resulting in chronic gastritis. Although this condition may be asymptomatic, some patients develop dyspeptic symptoms, *i.e.*, the so-called non-ulcer dyspepsia (NUD) [2]. In about 15% of infected patients, gastritis may progress further to severe gastric diseases, namely peptic ulcer disease (PUD) and gastric cancer (GC) [1], [3].

PUD is a multifactorial disease which leads to considerable patient morbidity and mortality [4]. It is considered to be a disease of adulthood being its progression highly dependent on environmental factors, such as the use of non-steroid anti-inflammatory drugs (NSAID), alcohol consumption, diet, smoking and stress. Some viral infections (*e.g*. cytomegalovirus and herpes simplex infections), Crohn's disease and syndromes where an increase in acid secretion occurs (*e.g.*, Zollinger-Ellison syndrome), may also contribute to the onset of PUD [4]. However, a long-term infection with *H. pylori* is considered as the major causative factor for PUD. This is easily demonstrated by the high number of patients infected with *H. pylori* among those who are affected by PUD, and by the fact that, with the successful eradication of the bacteria, non-NSAID-related PUD is healed and rarely recurs [2], [4]. PUD can be subdivided into duodenal ulcer (DU) and gastric ulcer (GU) diseases which are considered divergent. DU is basically a duodenal acid injury that results from acid hypersecretion due to infection of the antrum by *H. pylori* [2]. In contrast, GU is usually associated with acid hyposecretion that results from the gastric atrophy caused by a proximal spread of the infection, and hence, of inflammation [2]. Supporting differences in the pathogenesis of these two clinical outcomes, the prevalence of *H. pylori* infection is higher than 95% in DU cases and around 60% to 80% in GU [5], [6].

The development of PUD in *H. pylori* infected children is a very rare event and usually occurs soon after infection. In Portugal, for example, only about 2% of the estimated 40% of the Portuguese infected children suffer from this disease [7]. Besides genetic susceptibility [8], the aforementioned environmental factors should have a minor influence on the pathogenesis of PUD in infected children. Therefore, we believe that the virulence factors of the implicated strain must play a crucial role in the onset of PUD in children [2]. We have reported some data on genetic studies of clinical isolates which show the association of some bacterial genes with the development of PUD in infected children [9]–[12]. Indeed, in addition to the most well known virulence factors, namely the *cytotoxin-associated gene A* (*cagA*) gene, the “on” genotype (*i.e.*, functional) of the *outer inflammatory protein A* (*oipA*) gene, the s1/m1 allele of the *vacuolating cytotoxin gene* (*vacA*), and the *A2* allele of the *blood group antigen binding adhesin* (*BabA*) gene [1], [3], we found that *homB* and *jhp562* genes are closely associated with PUD in children and may be useful in determining the clinical outcome of infection [9]–[12]. HomB protein is an antigenic outer membrane protein which was shown to be involved in the host inflammatory response, inducing the secretion of interleukin-8 (IL8), and in the adhesion of *H. pylori* to gastric epithelial cells [9], [10]. The *jhp562* gene encodes a glycosyltransferase involved in the synthesis of lipopolysaccharide and may also be implicated in the regulation of Lewis antigen expression [12]. The presence of the triple genotype *cagA*, *jhp562* and *homB* in *H. pylori* strains provides a good discriminatory basis to distinguish PUD and NUD outcomes in infected children [12].

Motivated by these findings, we further characterized five *H. pylori* strains, isolated from Portuguese children with PUD, all positive for *cagA*, *jhp562* and *homB* genes. Their virulence profile was first compared to that of five other clinical isolates collected from children with NUD, by co-culture assays with the NCI-N87 cell line (American Type Culture Collection (ATCC) CRL 5822). We report here the differences that we found in the impact of these two groups of *H. pylori* strains on the viability, phenotype and cytoskeleton organization of the NCI-N87 cells. Moreover, in order to understand the molecular mechanisms that underlie their more pathogenic phenotype, we compared the proteomes of the ulcerogenic *H. pylori* strains with those of the NUD strains.

## Results

Ten *H. pylori* strains isolated from Portuguese pediatric patients, five suffering from NUD (mean age 10.2 years, range 7 to 14) and five other affected by PUD (mean age 11.8 years, range 10 to 15, having no other etiology for the disease), were carefully selected to be genetically homogeneous within the respective group (Table 1). The difference between the mean ages of these two groups was not statistically significant. According to their previously reported genotype [12], all PUD strains were positive for some important virulence factors associated with PUD in children. These include *cagA*, *vacA* s1 (*i.e.*, the toxic allele), *oipA* “on”, *homB* and *jhp562* (with the exception of strain 1846/05) genes (Table 1). In contrast, all of the NUD strains were *cagA*, *homB* and *jhp562* negative and carried the *oipA* “off” status and the *vacA* s2 (*i.e.*, the non-toxic allele) gene (Table 1).

**Table 1:** Characterization of the *H. pylori* strains included in the study and of the patients from whom each of them was isolated.

*H. pylori*						Patient		
Strain	*cagA*	*vacA*	*oipA*	*homB*	*jhp562*	Histologic Analysis	Age	Gender
**173/00**	−	s2	off	−	−	NUD	14	M
**207/99**	−	s2	off	−	−	NUD	7	F
**228/99**	−	s2	off	−	−	NUD	8	M
**655/99**	−	s2	off	−	−	NUD	11	M
**1786/05**	−	s2	off	−	−	NUD	11	M
**499/02**	+	s1	on	+	+	GU	11	M
**1089/03**	+	s1	on	+	+	DU	10	M
**1152/04**	+	s1	on	+	+	DU	10	M
**1198/04**	+	s1	on	+	+	DU	15	M
**1846/05**	+	s1	on	+	−	DU	13	M

All strains are indicated by their collection number. All patients were from Portugal. In columns *cagA*, *homB* and *jhp562*: + indicates strain containing the gene; − indicates strain lacking the gene. *vacA* column: s1 indicates strains containing the toxic allele of the gene; and s2, strain containing the non-toxic allele of the gene. *oipA* column: on indicates strains containing the functional allele of the gene; and off, strains containing the non-functional allele of the gene. NUD - non-ulcer dyspepsia; DU – duodenal ulcer; GU – gastric ulcer. M - Masculine; F - Feminine.

### Co-culture assays

**Figure 1:**
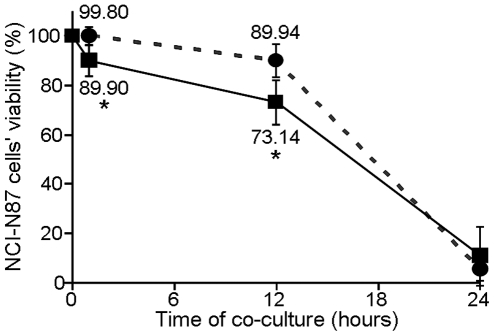
Impact of the two isogenic groups of pediatric *H. pylori* strains on NCI-N87 cells viability. The viability of NCI-N87 cells was assessed by the trypan blue exclusion assay after 1, 12 and 24 h of co-culture with a pool of five pediatric PUD-associated strains (▪ in full line) and a pool of five pediatric NUD-associated strains (• in dashed line). For normalization, the value of 100% corresponds to the viability of non-infected NCI-N87 cells. * indicates values that were determined as significantly different (*p*<0.05) between the viability of cells infected with PUD strains and that of cells infected with NUD-strains at the same time-point of co-culture. Symbols and error bars are means ± SD of the values at each point for 3 observations.

**Figure 2:**
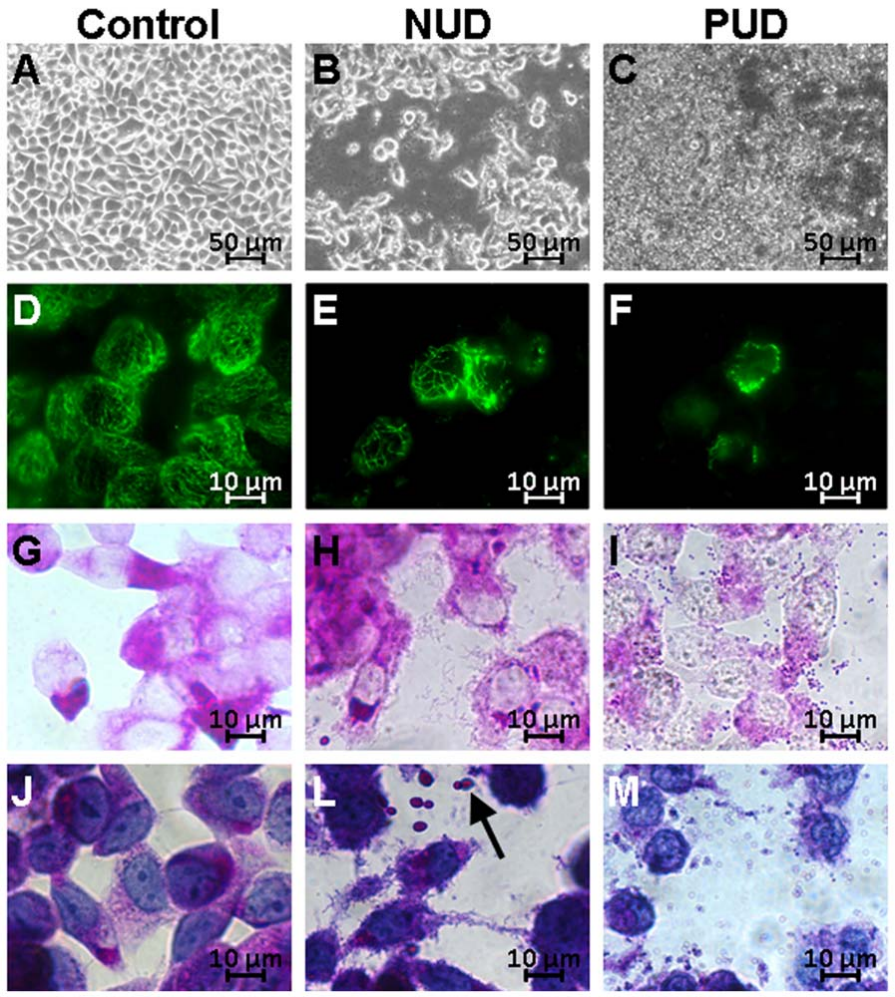
Impact of the two isogenic groups of pediatric *H. pylori* strains on the NCI-N87 cells morphology. NCI-N87 cells grown to 80–90% confluence were co-cultured for 24 h with the pool of five isogenic NUD-associated *H. pylori* strains (NUD) and in parallel with the pool of five isogenic PUD strains (PUD). The control corresponds to non-infected NCI-N87 cells. A), B), and C) light microscopy observations. D), E), and F) immunodetection of the microtubule network (green) (1∶1000 α-tubulin antibody plus a FITC conjugated secondary antibody). G), H), and I) PAS staining alone, and in J), L), and M) in conjugation with hematoxylin. Dark arrow indicates extracellular vesicles that reacted with PAS.

To clarify whether the virulence of the strains was the determining factor for the development of PUD in children from whom they were collected, we compared their impact on human gastric epithelial cells with that of NUD strains, by *in vitro* infection experiments. These experiments were carried out using the NCI-N87 cell line, known for its unique differentiation status as it is able to form coherent monolayers, expressing the correct cellular distribution of both *E*-cadherin and ZO-1 junction proteins [13]. Confluent NCI-N87 cells monolayers were thus incubated in parallel with two different pools of *H. pylori* strains, one including the five PUD associated strains, and another including the five NUD strains, both at a multiplicity of infection (MOI) of 100. Under our experimental co-culture conditions, the viability of NCI-N87 cells was clearly reduced over time with both pools of *H. pylori* strains (Figure 1). However, this effect was more pronounced in the first hours following infection with the pool of PUD-associated strains. Indeed, cell viability was significantly lower (*p*<0.05) in NCI-N87 cells infected with PUD-*H. pylori* strains at 1 h (89.90%±6.23) and 12 h post-infection (73.14%±8.96), compared to cells infected with NUD strains (99.80%±3.52 and 89.94%±6.70, respectively) (Figure 1). At 24 h post-infection, the viability of the epithelial cells was very low in both cases, 5.40%±4.51 and 10.78%±11.91, for cells infected with NUD and PUD strains, respectively (Figure 1). However, the morphological analysis of the remaining cells (Figure 2) showed dramatic differences in the impact of each pool of *H. pylori* strains. Indeed, light microscopy observations revealed much more pronounced cell damage in NCI-N87 cells infected with the pool of PUD-*H. pylori* strains (Figures 2A, 2B and 2C). Moreover, the pool of PUD strains caused the total destabilization of the NCI-N87 cells' microtubule network, as demonstrated by the immunocytochemical assays (Figures 2F), which seemed almost unchanged in cells infected with NUD-*H. pylori* strains (Figure 2E). In addition, epithelial cells infected with the PUD-*H. pylori* strains showed a drastic reduction in the cytoplasmic Periodic Acid Schiff (PAS) reactivity (Figure 2G, 2H and 2I), which was supported by the destruction of the cell membrane observed with hematoxylin and PAS double staining (Figure 2J, 2L and 2M). We also observed that the nuclear envelope of the NCI-N87 cells infected with the *H. pylori* strains associated with PUD was undisrupted, despite having a very irregular appearance, suggesting cell death by necrosis (Figure 2I and 2M). In contrast, NCI-N87 cells co-cultured with the NUD-associated *H. pylori* strains showed an accumulation of extracellular vesicles positive for PAS (dark arrow in Figure 2L) suggesting the expected exocytosis of intracellular mucus-containing vesicles, also observed in the non-infected control (data not shown). After 24 h of co-culture, NUD strains maintained their bacillary shape and were adherent to the epithelial cell surface (Figure 2H and 2L), however, PUD strains acquired a coccoidal shape (Figure 2I and 2M) which was already observed at 12 h post infection (data not shown).

### H. pylori proteome profile

**Figure 3:**
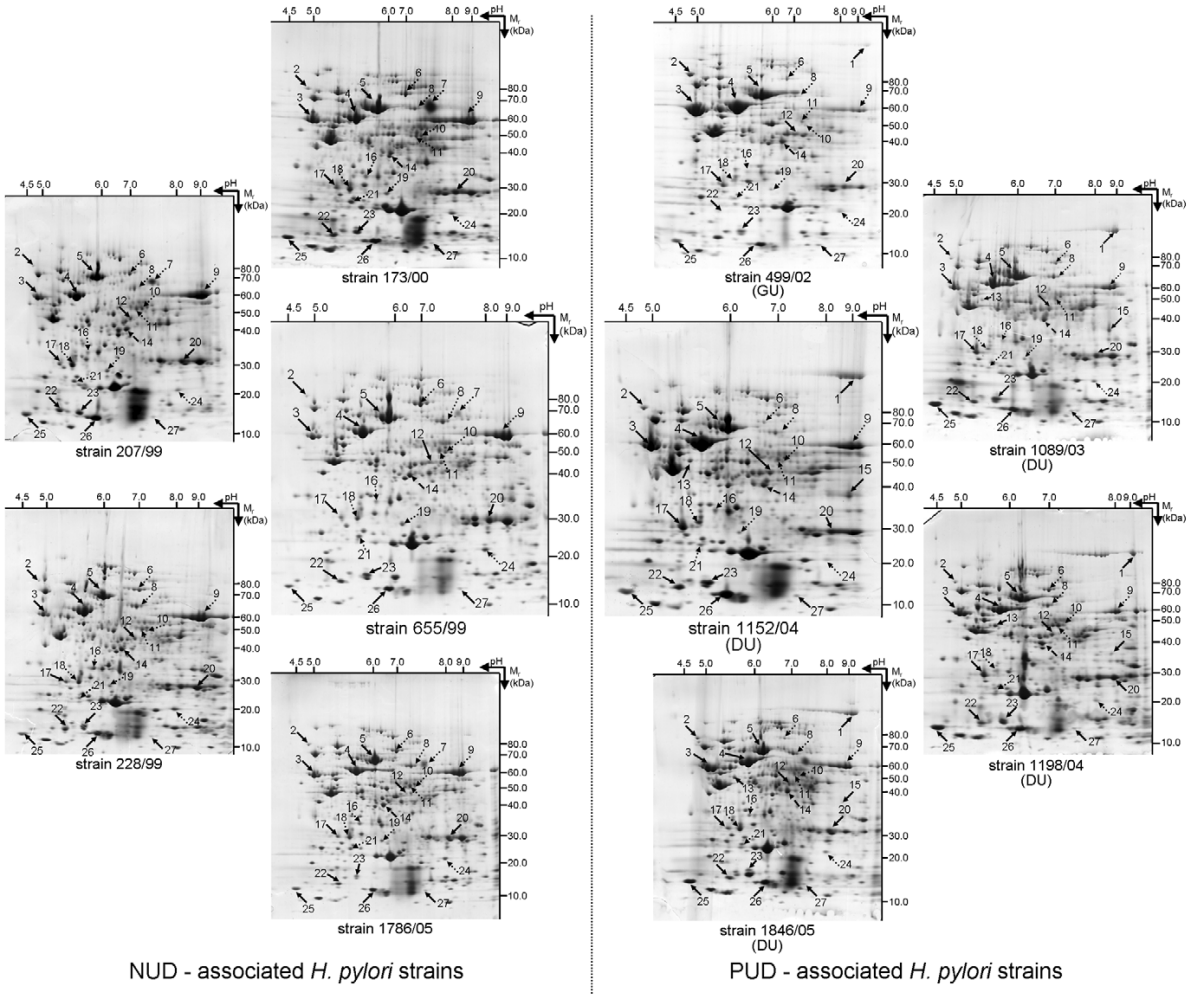
Profile of the soluble proteome of the 10 studied pediatric *H. pylori* strains. These include five strains associated with NUD (173/00, 207/99, 228/99, 655/99 and 1786/05), four from patients affected by DU (1089/03, 1152/04, 1198/04 and 1846/05) and a strain associated with GU (499/02). Proteins from the total biomass recovered from a 24 h grown plate were separated by 2DE, *i.e.*, first in a non-linear pH 3–11 and than in a 7–16% (w/v) SDS-PAGE. After CBB-staining, gels were analyzed using ImageMaster™ 2-D Platinum software. Black arrow indicates spots that were more abundant in PUD (DU+GU) strains when compared to NUD strains; Dotted arrow indicates spots that were less abundant in PUD (DU+GU) strains when compared to NUD strains; Dashed arrow indicates the spot that was shifted right in DU strains when compared to NUD strains. Spot numbers indicated in the 2DE maps are the same as used in Table 2.

Total soluble protein extracts from each of the 10 *H. pylori* strains were resolved by 2-dimensional gel electrophoresis (2DE), first in a non-linear pH range of 3–11 and then in a 7–16% (w/v) SDS-PAGE. After Coomassie Brilliant Blue (CBB) staining, the digitalized images of the gels were analyzed using the ImageMaster™ 2D Platinum software. Protein spots were automatically detected and manually corrected and the best resolved 2DE-gel, that of NUD-associated *H. pylori* strain 655/99, was used as the reference map during computer assisted analysis. After automatic matching, followed by a manual correction, gels were separated into two classes, NUD and PUD, according to the pathology with which the strain was associated. Gel-to-gel matching was confirmed by MS identification of matched spots from different 2DE gels. The 2DE maps obtained for each group of strains were found to be highly reproducible and consistent (Figure 3). No statistically difference was observed for the number of detected spots (on average 485±77 *vs.* 443±43 in NUD and PUD 2DE gels, respectively) between these two classes.

### Comparative proteomic analysis

In order to search for a protein signature for pediatric *H. pylori* PUD-associated strains, a computer-assisted analysis was performed by statistical evaluation of changes in the % Vol, *i.e.*, the normalized volume, for each protein spot within the two disease-associated classes of 2DE gels. As a result, irrelevant variations between images were eliminated. From this analysis, we observed that the proteome of the *H. pylori* strain 499/02, the unique strain associated with GU, was not fully consistent with the proteome of the other four strains included in the group of PUD related strains isolated from children with DU. Therefore, from this point onward the strain 499/02 was analyzed separately. Table 2 lists the identification by peptide mass fingerprinting (PMF) analysis and/or by comparison with 2DE databases [14] of 26 protein spots detected as being differentially expressed: 24 of them were statistical significant (*p*<0.05) between the NUD and DU classes of strains; and one spot detected at a different position within the 2DE-gels.

**Table 2:** Identification of proteins found to be differentially expressed in *H. pylori* strains from PUD patients (n = 5) compared to NUD patients (n = 5).

					Theoretical						% Vol					
Spot no.	Protein	Cellular function	ORF	Swiss-Prot/NCBI no.	Mw (kDa)	pI	MOWSE score		Peptides matched	Sequence coverage (%)	Average NUD ± S.D	Average DU ± S.D	Variation (DU/NUD)	*p value*		Variation (GU/DU)
1	CagA, Cytotoxin associated protein A	Induces abnormal proliferation; tight junctions disruption; cytoskeleton rearrangements	HP0547	gi|307135439	131	8.9	82	*	10/16	7	absent	1.558±0.487	**↑**	5.265E-08	*	**↓**
2	FlgE, Flagellar hook protein	Component of bacteria flagellum basal body	HP0870	Q9ZKY0	76	5.0	71	*	12/35	17	0.077±0.036	0.136±0.055	**↑**	2.099E-02	*	**↑**
3	FlaA, Flagellin A	Major component of bacteria flagellum	HP0601	P0A0S2	53	6.0	121	*	14/37	14	2.130±0.794	3.063±0.941	**↑**	4.706E-02	*	**↑**
4	HspB, Chaperone and heat shock protein	Protein folding/stress response	HP0010	B6JPA7	58	5.4	93	*	18/55	26	5.021±0.556	5.856±0.533	**↑**	7.801E-03	*	**-**
5	UreB, Urease beta subunit	Urea metabolism	P14917	P69996	62	5.6	Comparison to 2DE database				8.815±1.310	10.477±1.455	**↑**	3.551E-02	*	**↓**
6	HyuA, Hydantoin utilization protein A	Arginine and proline metabolism	HP0695	gi|108563105	79	6.4	105	*	24/100	20	0.503±0.128	0.204±0.110	**↓**	4.079E-04	*	**-**
7	Unknown	—	—	—	—	—	—				1.094±0.702	absent	**↓**	2.706E-04	*	**-**
8	RpsA, 30S Ribosomal protein s1	Translation process	HP0399	P56008	63	6.3	Comparison to 2DE database				0.126±0.029	0.085±0.022	**↓**	1.141E-02	*	**-**
9	KatA, Catalase	Detoxification	HP0875	P77872	59	8.7	161	*	21/48	37	4.833±0.962	2.669±0.621	**↓**	1.292E-04	*	**-**
10	CysS, Cysteinyl-tRNA synthetase	Translation process	HP0886	B5Z7P5	53	6.0	28		4/23	8	0.152±0.091	0.027±0.024	**↓**	2.615E-03	*	**-**
11	AspA, Aspartate ammonia-lyase	Catalyzes the deamination of aspartate originating fumarate	HP0649	gi|207108618	35	8.5	58		5/23	10	0.396±0.097	0.252±0.089	**↓**	9.261E-03	*	**-**
12	Pgk, Phosphoglycerate kinase	Glucose metabolism	HP1345	gi|261838924	44	6.3	86	*	10/33	27	0.213±0.064	0.303±0.069	**↑**	2.525E-02	*	**↑**
13	HPAG1_1081, Hypothetical protein	Putative function in cell shape	HP1143	gi|108563506	51	5.5	77	*	11/23	15	absent	0.200±0.072	**↑**	3.577E-07	*	absent
14	EF-Ts, Elongation factor Ts	Translation process	HP1555	B6JP47	40	6.2	76	*	7/13	16	0.606±0.101	0.613±0.185	**↔**	9.206E-01		normal position
15	Putative aldo-keto reductase	Catalyze the reduction of aldehydes and ketones to the corresponding alcohol product	HP1193	gi|254779741	37	6.7	86	*	8/18	22	0.015±0.040	0.147±0.224	**↑**	7.418E-03	*	**↓**
16	HELPY_0944, Hypothetical protein	Motility	HP0958	gi|254779556	30	5.6	99	*	13/46	41	0.231±0.080	0.165±0.026	**↓**	8.453E-02		**-**
17	HP_1588, UPF0174 protein	Unknown	HP1588	O26107	29	5.5	85	*	8/17	15	0.322±0.123	0.563±0.373	**↑**	2.293E-03	*	**↓**
18	HP_1588, UPF0174 protein	Unknown	HP1588	O26107	29	5.5	90	*	10/30	28	0.853±0.192	0.399±0.167	**↓**	5.076E-04	*	**-**
19	ScoA, Succinyl-CoA-transferase subunit A	Citric acid cycle	HP0691	P56006	26	5.8	41		3/7	11	0.199±0.051	0.052±0.053	**↓**	3.739E-05	*	**-**
20	UreA, Urease subunit alpha	Urea metabolism	HP0073	Q9ZMZ4	27	8.5	145	*	12/26	43	0.798±0.078	1.082±0.214	**↑**	3.314E-03	*	**↓**
21	ScoB, Succinyl-CoA-transferase subunit B	Citric acid cycle	HP0692	gi|208434607	22	5.4	104	*	10/33	33	0.242±0.066	0.149±0.061	**↓**	1.070E-02	*	**-**
22	AroQ, 3-dehydroquinate dehydratase	Aromatic aminoacid biosynthesis	HP1038	B6JKY5	19	5.1	77	*	8/36	35	0.520±0.108	0.622±0.077	**↑**	5.503E-02		**↑**
23	Pfr, Ferritin	Iron storage protein	HP0653	Q9ZLI1	19	5.4	40		4/23	23	0.728±0.295	1.185±0.193	**↑**	4.285E-03	*	**-**
24	Porγ, Pyruvate flavodoxin oxidoreductase, gamma subunit	Decarboxylation of pyruvate in acetyl-CoA	HP1108	gi|15645722	21	8.5	88	*	10/33	36	0.313±0.086	0.141±0.033	**↓**	1.133E-04	*	**-**
25	FldA, Flavodoxin	Electron transport process	HP1161	O25776	17	4.5	73	*	5/14	42	0.657±0.277	1.259±0.430	**↑**	6.800E-03	*	**-**
26	NapA, Neutrophil activating protein A	Protects DNA from oxidative damage; activates neutrophils, mast cells and monocytes	HP0243	gi|7188724	17	5.7	103	*	16/100	79	1.350±0.567	1.933±0.360	**↑**	2.996E-02	*	**-**
27	HPG27_1480	Putative function in cell shape	HP1542	gi|208435422	11	5.6	48		3/13	35	0.065±0.016	0.088±0.007	**↑**	4.953E-03	*	**-**

“Comparison to 2DE databases” indicates spots for which identification was done by finding the corresponding spot on the standard *H. pylori* 2DE gel from the database [14]. All of the others were identified by PMF analysis. For each spot the average of the % Vol ± S.D. is indicated. In the Variation (DU/NUD) column: ↑, proteins for which the average of the % Vol of spots on DU 2DE maps was higher than that on NUD 2DE maps; ↓, proteins for which the average of the % Vol of spots on DU 2DE maps was lower than that on NUD 2DE maps; ↔, proteins that were shifted right in the DU 2DE-maps regarding their position on the NUD 2DE-maps. In the Variation (GU/NUD) column: ↑, proteins for which the average of the % Vol of spots on GU 2-DE maps was higher than that on the DU 2DE maps; ↓, proteins for which the average of the % Vol of spots on GU 2DE maps was lower than that on DU 2DE maps;  = , proteins showing the same pattern of expression observed for the *H. pylori* strains associated with DU. Asterisks indicate statistical significant results (*p*<0.05). ^1^Indicates the identified ORF in the 26695 reference strain.

These include 15 proteins which were highly abundant in DU-associated *H. pylori* strains (solid arrows in Figure 3; upward arrows in Table 2), when compared to NUD strains: flagellar hook protein (FlgE, spot 2) and flagellin A (FlaA, spot 3), both components of the bacteria's flagella; the heat shock protein B (HspB, spot 4), a homolog of Hsp60 involved in protein folding; both beta and alpha subunits of urease (UreB, spot 5, and UreA, spot 20), which catalyzes the hydrolysis of urea into ammonia and CO_2_; phosphoglycerate kinase (Pgk, spot 12), 3-dehydroquinate dehydratase (AroQ, spot 22) and flavodoxin A (FldA, spot 25), involved in bacteria metabolic cycles; putative aldo-keto reductase (spot 15), a protein of the cell detoxification system; an isoform of UPF0174 protein HP_1588 (spot 17), a protein whose function is unknown; the non-heme iron-containing ferritin (Pfr, spot 23), involved in iron metabolism; the virulence factor neutrophil activating protein (NapA, spot 26); and the HPG27_1480 protein (spot 27), homologous to the HP1542 protein which has a putative function in cell shape [15]. Interestingly, a thirteenth protein, the hypothetical protein HPAG1_1081 (spot 13), was detected exclusively in the DU- associated *H. pylori* strains. This protein is a homolog of HP1143, another protein with a putative function in cell shape [16]. Further experiments are needed to clarify whether the expression of this protein is unique to the DU strains, or it also occurs in NUD and GU strains but at levels below our detection threshold. As previously shown, only the PUD-*H. pylori* strains were *cagA* positive and this was confirmed by the total absence of CagA protein in the 2DE maps of the NUD-strains. This protein corresponds to spot 1 in the PUD-2DE maps (Figure 3; Table 2).

Amongst the group of differentially expressed proteins, we found the following 11 protein spots to be less abundant in DU-associated *H. pylori* strains (dotted arrows in Figure 3; downward arrows in Table 2), when compared with NUD strains: hydantoin utilization protein A (HyuA, spot 6), aspartate ammonia-lyase (AspA, spot 11), succinyl-CoA-transferase (SCOT) subunits A and B (ScoA and ScoB, spots 19 and 21, respectively), pyruvate flavodoxin oxidoreductase, gamma subunit (Porγ, spot 24), proteins involved in bacteria metabolic cycles; ribosomal protein S1 (RpsA, spot 8) and cysteinyl-tRNA synthetase (CysS, spot 10), proteins involved in protein biosynthesis; catalase (KatA, spot 9), involved in the cell detoxification system; HELPY_0944 (spot 16), homologous to HP0958, which is a protein with a putative function in cell motility; and another isoform of UPF0174 protein HP_1588 (spot 18), a protein whose function is unknown. Spot 7, which we were unable to identify, was only detected in NUD strains, with the exception of the *H. pylori* strain 228/99. As before, we cannot say that the expression of this protein is completely absent in DU strains, rather that its expression levels were below our detection threshold.

Besides differences in protein expression between these two groups of strains, we detected a difference in the position of the protein spot 14 within the 2DE-gel (Figure 3) that was identified as being elongation factor Ts (EF-Ts) (Table 2). Indeed, in the 2DE-maps of the DU strains, this protein spot was shifted to the right relative to its position in the NUD 2DE-maps (Figure 3).

Interestingly, we found differences in the abundance of some of these protein spots in GU-associated *H. pylori* strain 499/02, when compared to that of the DU strains (Figure 3; Table 2). Showing the same tendency of that observed in DU strains compared to NUD strains, we found FlgE (spot 2), FlaA (spot 3), Pgk (spot 12) and aroQ (spot 22). Showing the opposite tendency of that observed for DU strains compared to NUD strains, and with abundance levels similar to those observed in the latter group of strains, we found UreB (spot 5), HPAG1_1081 (spot 13), putative aldo-keto reductase (spot 15), one isoform of the HP_1588 (spot 17), and UreA (spot 20). CagA (spot 1) was less abundant in the GU strain (Table 2). All of the other protein spots followed the same pattern of expression observed for the *H. pylori* strains associated with DU (Table 2).

Although the MOWSE score in the MS identification did not reach statistical significance (*p*>0.05) for the CagA (spot 1), CysS (spot 10), AspA (spot 11), ScoA (spot 19), Pfr (spot 23) and HPG27_1480 (spot 27) protein spots, we are confident in our results because the same identification was obtained for equivalent spots from different 2DE gels and it also matched with the identification in the 2DE *H. pylori* database [14].

### Motility assay

**Figure 4:**
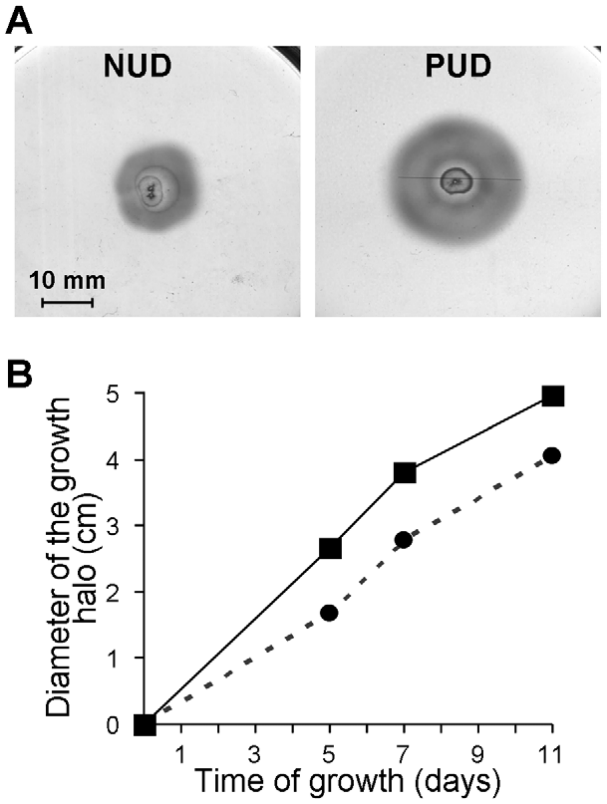
Motility of the two isogenic groups of pediatric *H. pylori* strains in study. The five strains of each class, recovered from 24 h grown plates, were pooled together, inoculated on semi-solid BHI broth −5% FBS medium plates and incubated for 11 days at 37°C under microaerophilic conditions. A) The growth halo observed at day 5 for the pool of PUD and of NUD strains. B) Variation of the diameter of the growth halo along the time (▪ in full line refers to PUD strains and • in dashed line refers to NUD strains). Symbols are means of the values at each point for 2 observations.

As there were proteins related somehow to bacteria motility included in the group of proteins which were expressed differentially between NUD and PUD strains, we further evaluated whether this was translated into differences in motility. Accordingly, the pools of five NUD strains and of five PUD strains under study were inoculated in an agar motility medium and, at 5, 7 and 11 days the diameter of the growth halo was measured (Figure 4). In agreement with the higher abundance of FlgE (spot 2), FlaA (spot 3) in PUD strains (both in DU and GU strains), the *H. pylori* strains associated with PUD in children showed bigger growth halos (Figure 4), indicating that they have higher motility than the strains associated with NUD. These experiments were carried out twice, leading to consistent results.

## Discussion

The development of PUD in children is a rare consequence of infection by *H. pylori*. Environmental factors should have a minor influence on its pathogenesis since pediatric PUD develops soon after infection. Its dependence on the genetic susceptibility of these children is still poorly understood. The expression of sialyl-Lewis (x) antigens in gastric epithelial cells in association with *H. pylori*-dependent DU in pediatrics was recently reported. However, in contrast to the aberrant expression found in adults patients, the gastric mucosa of these children presents a normal pattern of expression and glycosylation of MUC6 or MUC2 [8]. Moreover, the susceptibility associated to male gender has been described and the hypothesis that female hormones, before the onset of the menopause, somehow protect against the development of PUD has been raised [17], [18]. In agreement, we have recently reported that pediatric PUD is significantly more frequent in boys than in girls (63.6% *vs* 36.4%, *p*<0.025) when analyzing a large cohort of 1,115 Portuguese children [19]. Notwithstanding, the involvement of more pathogenic strains is surely a key factor in pediatric *H. pylori*-dependent PUD, as was clearly demonstrated by our previous reported data [9]. In order [10]–[12] to better characterize the virulence of such strains, we studied a group of five *H. pylori* strains isogenic for important virulence factors associated with pediatric PUD, namely *cagA*, *vacA* s1 allele, *oipA* “on”, *homB* and *jhp562* (with the exception of the strain 1846/05) genes (Table 1). These were compared to another group of five strains, isolated from Portuguese children presenting NUD, all negative for those virulence factors (Table 1).

In agreement with the more pathogenic behavior of PUD *H. pylori* strains, our co-culture assays showed that, indeed, the five PUD-associated strains, when pooled together, caused a more pronounced reduction in the viability of NCI-N87 cells than the pool of five NUD-associated strains (Figure 1). In contrast to NCI-N87 cells in co-culture with NUD strains, the remaining epithelial cells after 24 h of co-culture with PUD strains presented a dramatic destabilization of the microtubule network (Figure 2F) and a decrease in the cytoplasmic reactivity to PAS (Figure 2I), suggesting lower amounts of mucins . These results are consistent with the decreased levels of mucins observed in gastric biopsies of infected adult patients with PUD and in cell lines in co-culture with *H. pylori* strains positive for both *cagA* and *vacAs1* genes [20], [21]. Double staining with PAS and hematoxylin showed that *H. pylori* PUD strains induce the destruction of the cytoplasmic membrane of some host cells, leading them to release their cytoplasmic content (Figure 2M). This is similar to necrosis, a required step in the PUD pathogenesis process. Probably due to changes in environmental conditions caused by massive cell death, PUD strains acquired a coccoidal form (Figures 2I and 2M). This phenotype was already observed at 12 h of co-culture.

**Figure 5:**
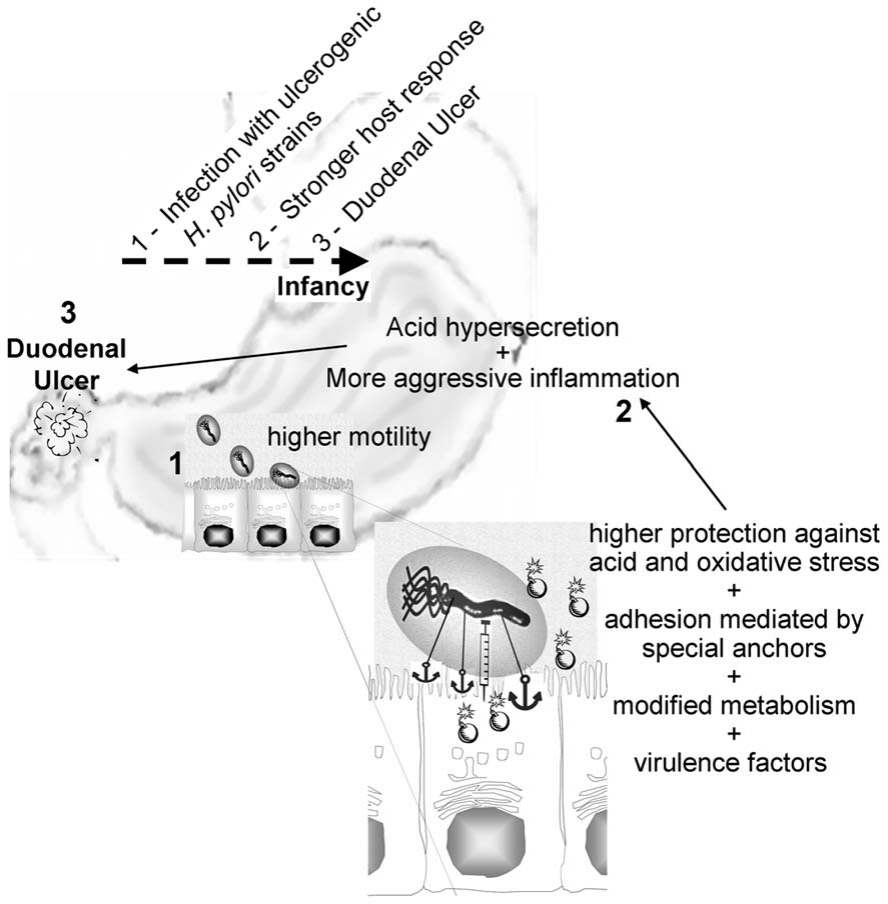
Schematic diagram of the DU development in children upon *H. pylori* infection. The virulence of the pediatric PUD-associated *H. pylori* strains results from a synergy between their natural ability to better adapt to the hostile human stomach and their virulence factors. Adaptation is ensured by: higher motility (↑ FlaA, ↑ FlgE, ↓ HELP_0944, ↑ HPAG1_1081, and ↑ HPG27_1480), higher ability to survive under low pH conditions (↑ UreA and B and ↑ putative aldo-keto reductase), better antioxidant defenses against inflammation (↑ Pfr, ↑ NapA and ↓ KatA), modified metabolism (↓ HyuA, ↓ AspA, ↑ Pgk, ↓ ScoA and B, ↑AroQ, ↓ Porγ, ↑ FldA, ↓ RpsA and ↓ CysS), adhesion (indicated by a anchor) mediated by “on” OipA and HomB. Virulence factors (indicated by a bomb): cagA and VacAs1. 1 – Infection; 2 – Host response (acid hypersecretion and inflammation); 3 – duodenal acid injury (Duodenal Ulcer, indicated by an explosion symbol). Dotted arrow - Time line.

Taking advantage of our pediatric strains, we decided to evaluate the proteome of each of the 10 Portuguese *H. pylori* strains. Their proteome profiles were consistent (Figure 3) and comparable with proteome profiles in databases [14]. Moreover, 27 proteins were found to be differentially expressed between PUD and NUD strains (Table 2). It should be stressed that these differences in protein expression profile are intrinsic to the bacteria and were not induced by the environment. In fact, despite having experienced completely different environmental conditions *in vivo*, in this study they were grown under the same controlled conditions which did not fully resemble their natural niche. An important observation was noted for the group of PUD-associated strains: the strain 499/02 presented unique features which required special attention given that it was isolated from a child with GU and the other four were associated with DU. Although these diseases share important molecular mechanisms in their pathogenesis, they are characterized by different patterns of colonization, gastritis and gastric acid secretion [2]. Our results suggest that the ability to induce one of these situations is reflected in the proteome of the implicated *H. pylori* strains and should be investigated further. Thus, the strain 499/02 was considered separately in the subsequent analysis. Figure 5 resumes the following discussion of the differentially expressed proteins grouped according to their known function. We were not able to identify spot 7 but its role should be important role as it was absent from the proteome of all PUD strains.

### Motility

For efficient colonization of the gastric niche, *H. pylori* depends on its ability to swim [22]. The results of the five pediatric strains associated with PUD (Figure 4) show higher levels of FlgE (spot 2) and FlaA (spot 3) compared to NUD strains, justifying their enhanced motility. We must stress that spot 3 may result from the overlap of FlaA and FlaB (flagellin B), another component of the *H. pylori* flagellum filament, both having equal p*I* and similar molecular weight (53 and 54 kDa, respectively). If that is the case, our results reflect a change in the abundance of both FlaA and FlaB.The abundance of HELPY_0944 protein (spot 16) was decreased in the five PUD strains compared to the NUD strains. Despite its homology with the protein HP0958 (*H. pylori* strain 26695), these results point to a different cellular function for these two proteins. Contrary to our data on the expression of FlgE, FlaA (and FlaB) and HELPY_0944 proteins, *HP0958* mutants result in non-flagellated strains, showing a total abrogation of *flgE* and *flaB* transcription [23], [24] and very low levels of FlaA [25]. In fact, HP0958 protein is known to induce the transcription of *flgE* and *flgB* through stabilization of the RNA polymerase sigma factor RpoN [23], [24] and the synthesis of FlaA in an RpoN-independent manner [25].

Besides its polar flagella, *H. pylori*'s spiral morphology is a requirement for its motility. In line with this, HPAG1_1081 (spot 13) was exclusively detected in DU-*H. pylori* strains. HPAG1_1081 is a homolog of HP1143, a coiled-coil protein with a predicted intermediate filament-like function [26], thus contributing to the morphology maintenance [16]. HPG27_1480 protein (spot 27), another protein found more abundantly in all PUD strains, is homolog to HP1542, a protein involved in spiral shape maintenance [15].

### Antioxidant system

For a sustained infection of the inflamed gastric mucosa, characterized by the presence of large amounts of reactive oxygen species (ROS), *H. pylori* depends on the concerted activity of several proteins for its antioxidant defense [27]. Here we report higher levels of Pfr (spot 23) and NapA (spot 26) proteins and lower levels of KatA (spot 9) for the PUD strains.

Pfr is an essential protein for *H. pylori* which serves as a storage of iron in a bioavailable form [28]. Thus, besides protecting the bacterium against oxidative stress caused by the iron-mediated Fenton reaction in the presence of excessive amounts of this ion, Pfr also guarantees the necessary amount of iron for the normal metabolism of the cell under iron-limited conditions [29]. Like Pfr, NapA is capable of sequestering iron under oxidative stress conditions and these two share a protective role of the bacterial DNA [30]. NapA is, however, better known for its role in mediating the adhesion of *H. pylori* to host mucins and its ability to recruit neutrophils and monocytes to the infected gastric mucosa, inducing the secretion of chemokines [31]. According to the literature, abnormally high levels of NapA are expressed in mutant strains lacking KatA. Instrumental in catalyzing the decomposition of H_2_O_2_ into H_2_O and O_2_ [27], this enzyme has been described as being essential to the survival of *H. pylori* in a sustained long-term inflammation [27]. We therefore propose that the NUD strains analyzed in this study are prepared to resist in a long-term but less aggressive inflammation. However, the five pediatric strains associated with PUD are able to survive an acute inflammation that results in the early development of PUD.

Two isoforms of HP1588 protein (spots 17 and 18) were also differentially expressed between PUD and NUD strains. The exact function of this protein is not known although some studies indicate a potential role in stress resistance, as it is over-expressed under acidic [32] or iron starvation conditions [33].

### Acid Resistance

Urease is a key enzyme in the colonization and persistence of *H. pylori*, playing a central role in the bacteria resistance to gastric acidity by catalyzing the hydrolysis of urea into ammonia and CO_2_. Ammonia is, however, cytotoxic to the host cells, causing their necrosis. This multimeric protein of approximately 1100 kDa is composed of twelve small subunits, UreA (27 kDa), and twelve large subunits, UreB (62 kDa) [34]. In agreement with our previous findings [35], urease (UreA plus UreB) was one of the most abundant proteins in the proteome of all of the *H. pylori* strains in the current study. However, the strains collected from children with DU had higher levels of both UreA and UreB, than either the NUD or the GU strains. Higher levels of HspB (spot 4), a protein which functions as an extracellular chaperonin for urease [36] among other key roles, were observed in all PUD strains.

According to the literature, excessive urease activity under neutral conditions is lethal to the bacteria [34]. Considering that no difference was detected neither in the growth rate nor in the urease activity among these pediatric strains at pH 7 (results not shown), we hypothesize that the highest amount of urease produced by the DU strains does not reflect a higher activity *per se*. Instead, urease activity must be acid–dependent and thus, once in their natural niche, DU strains are better protected against a sudden drop in pH and/or in Ni^2+^ concentration which explains their ability to survive under abnormally acidic conditions [34]. Moreover, since urease has also been proposed to play a role in adherence to gastric mucins [37], again in a acid dependent manner [38], higher levels of urease may facilitate the processes of colonization and infection, contributing to the ulcerogenic phenotype of DU strains.

Lastly the putative aldo-keto reductase (spot 15) was also found to be more abundant in DU strains. It was described as an essential enzyme for growth at acidic pH [39], and is involved in the removal of toxic aldehydes and ketones of the cell. Again, the abundance of this protein in the proteome of the GU-associated strain 499/02 was equivalent to that in NUD strains, further corroborating the idea that only the DU-associated pediatric *H. pylori* strains are prepared to face a hyper-acidic environment.

### Metabolism

Among the proteins expressed differentially between NUD and PUD strains, there were 10 proteins involved in the metabolism of the bacterium.

A lower abundance of HyuA (spot 6), the enzyme responsible for the conversion of N-methyl hydantoin to N-carbamoyl sarcosine, was observed in all PUD-associated *H. pylori* strains. Not much is known about the role of this protein in *H. pylori* but this was described as a key step in the metabolism of creatine in other bacteria [40], a final pathway in the urea cycle and amino acid metabolism. Whether this may favor the endogenous production of urea we do not know and it should be investigated. In line with our findings, other authors have indicated HyuA as a biomarker for GC, since positive antibodies against it were found in sera from patients suffering with GC but not with PUD [41], [42]. Interestingly, AspA (spot 11), the enzyme that by catalyzing the deamination of Asp to fumarate ensures the aneuplerotic replenishment of the latter in Krebs cycle [43], [44], was in low abundance in all PUD strains compared with NUD strains. Asp and fumarate link the Krebs cycle to the urea cycle [45], therefore, we may suppose that in the former group of strains, Asp is primarily involved in the urea cycle with the concomitant production of urea. However, as *H. pylori* uses amino acids as a primary source of carbon, nitrogen and energy, with Asp being one of the eight most consumed amino acids [46], we cannot rule out that in PUD strains, Asp is used in the biosynthesis of Asp-derived amino acids. Indeed, the metabolism of amino acids was recently connected to virulence, colonization and stress resistance of *H. pylori* [46]. In *H. pylori*, the Krebs cycle is characterized by the absence of succinate dehydrogenase [44]. Although controversial, the SCOT complex which catalyzes the conversion of succinyl-CoA to succinate in an acetoacetate-dependent manner was proposed to substitute the missing enzyme [47]. Here *H. pylori* strains isolated from children with PUD presented lower levels of both subunits of the SCOT-complex, ScoA and ScoB (spots 19 and 21, respectively) which suggests a down-regulation of its catalytic activity.

Since *H. pylori* is a microaerophilic organism, the initiation of the Krebs cycle, *i.e.* the oxidative descarboxylation of pyruvate is catalyzed by an oxygen sensitive enzyme, pyruvate flavodoxin oxidoreductase (POR), instead of the aerobic pyruvate dehydrogenase or the strictly anaerobic pyruvate-formate lyase. Although its stoichiometry is unknown, POR is composed of four subunits, all essential for the *in vitro* viability of *H. pylori*. These are encoded by the *porCDAB* operon [48]. In this reaction, the FldA protein is the electron acceptor [43], [44], [49], a low potential one, which in turn is re-oxidized by the flavodoxin-quinone reductase (FqrB), generating NADPH. *In vitro* experiments have shown that POR is the rate limiting enzyme in this pathway [50]. The reverse reaction of this POR-FldA-FqrB-NADP reductase complex is believed to be important in the fixation of CO_2_, which is essential for replenishing pyruvate consumed by gluconeogenesis [50]. Interestingly, we found significantly lower levels of one isoform of the PORγ subunit (spot 24), encoded by the *porC* gene, in the pediatric PUD-associated *H. pylori* strains compared to the NUD strains. As far as we known, the role of PORγ subunit and its isoforms in the POR complex is still unclear. However, because of the observed higher levels of FldA (spot 25), we interpret our findings as PUD strains having a more functional POR. In this case, we would expect a higher production of NADPH and acetyl-CoA in PUD strains. Further experiments are needed to clarify this. Moreover, the Pgk protein (spot 12), a glycolytic/gluconeogenic enzyme which catalyzes the reversible conversion of 1,3-biphosphoglycerate into 3-phosphoglycerate [44], was found to be more abundant in the DU-associated strains and even more abundant in the GU related *H. pylori* strain.

Chorismate, a precursor of aromatic amino acids, is formed from phosphoenol pyruvate and erythrose 4-phosphate by means of seven enzymes [44]. AroQ (spot 22), an enzyme that catalyzes the conversion of 3-dehydroquinate into 3-dehydroshikimate which is a key step in chorismate synthesis, showed an augmented expression in DU-associated *H. pylori* strains and, again, an even higher expression in the GU strain. This is suggestive of an enhancement in aromatic amino acid biosynthesis in DU strains compared to NUD strains and even more pronounced in the GU strain over all of the other [51].

In *H. pylori*, amino acids in excess of those needed for protein synthesis cannot be stored and are catabolised. Our data point out that this ability is potentiated in the pediatric PUD-*H. pylori* strains. In fact, lower levels of both RpsA (spot 8), one of the 21 ribosomal proteins of the small 30S subunit [44], and aminoacyl-tRNA synthetase CysS (spot 10) were observed for these strains when compared to NUD strains. These data suggest a down-regulation of translation in general saving amino acids for degradation. If that is the case, the aforementioned higher levels of HspB registered for PUD strains would also play an important role in avoiding protein misfolding. The elongation factor EF-Ts (spot 14), also involved in translation, showed a shift in 2DE gels of DU strains, suggesting a post-translational modification which may influence its activity. In fully agreement with these results, two studies reported that under stress, nutritional or low pH conditions, the stringent response of *H. pylori* included the down-regulation of ribosomal genes and of aminoacyl-tRNAs and, in contrast, the enhancement of amino acid biosynthesis [44], [52]. The beauty of the data presented here is that are not induced by stress conditions but, as mentioned above, are intrinsic to PUD strains, making them much more adapted and, therefore, virulent.

### Virulence factors

As already mentioned, all of the *H. pylori* strains associated with PUD in children that we have analyzed carried the genes of some well known virulence factors (Table 1) from which we could only detect the expression of *cagA*. CagA (spot 1 in our 2DE gels) which is known to induce abnormal proliferation, disruption of tight junctions, cytoskeleton rearrangements and IL-8 secretion in the host cells [53], was less abundant in the GU strain when compared to the other four DU associated strains.

It was important to disclose whether the special features of the proteome profile of the PUD-*H. pylori* strains of this study were linked to their *cagA*/*vacA*s1 positive genotype. For that, we checked the abundance of the aforementioned 26 proteins in the proteome of ten other Portuguese *H. pylori* strains isolated from children and adult patients with NUD and from adults patients with PUD and GC (five *cagA/vacA*s1 positive and five others negative for these virulence factors), all currently under study in our lab. Of those proteins, only HspB and Pfr showed a pattern of abundance in the comparison between *cagA* positive and negative strains (data not shown) similar to that presented when comparing pediatric PUD and NUD strains (data from this study). This supports our hypothesis that the pediatric *H. pylori* strains associated with PUD present a specific protein signature which provides them a natural ability of adaptation to the human stomach. This profile combined with the expression of virulence factors (*cagA*, *vacA*s1, *oipA* “on” status, *homB* and *jhp562*) appears to be responsible for their enhanced virulence. Asian isolates which present marked differences in their genetic background compared to European strains, namely being nearly all positive for *cagA*, are an interesting group to study in the future.

### Conclusion

The data that we report here clearly show that the pediatric ulcerogenic *H. pylori* strains in our study share a particular proteome profile that, in addition to the well established virulence factors, provides them with higher motility. They also highlight for the strains' better antioxidant defenses and metabolism favoring the biosynthesis of aromatic amino acids, perhaps to be used as source of energy. Additionally, DU strains are apparently better fitted than all the other studied strains to survive under low pH conditions, which may justify their survival following acid hypersecretion which is characteristic of this disease. We believe that the virulence of pediatric ulcerogenic strains is strongly dependent on the synergy of their well established virulence factors and their better adaptation to the natural niche. Despite the relevance of these data, further research is required to determine their biological meaning under stressful conditions (acidic, oxidative and nutrient limited conditions). Moreover, host susceptibility should be evaluated in order to clarify its role in the pathogenesis of pediatric DU. Finally, it will be important to characterize all of the differences in the proteome profile of DU- and GU-associated *H. pylori* strains, to better understand the divergence of these diseases.

## Methods

### Bacteria and cell growth conditions

A total of 10 *H. pylori* strains isolated from children attending the pediatric gastroenterology units in the Lisbon area (Portugal) for upper diagnostic gastrointestinal symptoms were analyzed. These included five patients presenting NUD and five others with PUD (one GU and four DU) (Table 1). None of the children had received anti-*H. pylori* antibiotic or anti-secretory therapy prior to endoscopy. All of the children suffering from PUD had no other etiology for the disease. These strains belong to the collection of bacterial strains of the Department of Infectious Diseases of the National Institute of Health Dr. Ricardo Jorge, in Lisbon, Portugal. Bacteria were grown in *H. pylori* selective medium (Biogerm, Maia, Portugal) at 37°C in a microaerobic environment (Anoxomat®, MART Microbiology BV, Drachten, The Netherlands) for 24 h. For motility and co-culture assays, a pool of the five NUD strains and a pool of the five PUD strains were prepared by mixing biomasses recovered from 24 h grow plates of each strain (equal amount of each strain).

For co-culture assays, the NCI-N87 (ATCC CRL 5822) cell line was grown at 37°C with 5% CO_2_ and 99% humidity in Dulbecco's modified Eagle's medium (DMEM/F12) (Invitrogen, Life Technologies, Carlsbad, CA, USA) supplemented with 10% (v/v) of heat inactivated (56°C for 30 min) fetal bovine serum (FBS) (Invitrogen).

### Co-culture assays

A pool of the five NUD-associated strains and a pool of the five PUD-associated strains were prepared in NCI-N87 cell growth medium, and diluted to a final concentration of 1×10^8^ CFU/mL. NCI-N87 cells grown on 8-well chamber slides (Nalge Nunc, Roskilde, Denmark) for immunocytochemistry assays or on 24 multi-well plates (Nalge Nunc) for cellular viability determination, until 80 to 90% confluence were rinsed twice with phosphate-buffered saline (PBS) (Invitrogen) and fresh growth medium was added. Bacterial pools were then added at a MOI of 100 and the plates were maintained under NCI-N87 cell growth conditions. Non-infected NCI-N87 cells were used as a control. At 1, 12 and 24 h post-infection, cells were analyzed by light microscopy and stained using PAS (Sigma-Aldrich Co., St. Louis, MO, USA) and hematoxylin (Sigma-Aldrich). Cell viability was determined using the classical trypan blue exclusion test (Sigma-Aldrich).

### Immunocytochemistry analysis

After co-culture with the pools of *H. pylori* strains, NCI-N87 cells were rinsed twice with cold PBS and fixed for 30 min at 4°C in a 4% (v/v) formaldehyde (Sigma-Aldrich) and 3.7% (w/v) sucrose (Merck, Darmstadt, Germany) solution, in PBS. After two washes with PBS, cells were permeabilized for 15 min with 0.2% (v/v) Triton X-100 (Sigma-Aldrich) in PBS at room temperature (RT), washed three times more with PBS and blocked with 1% (w/v) bovine serum albumin (BSA) in PBS for 30 min at RT, prior to incubation for 2 h at 37°C with the anti-α-tubulin (clone DM1A) (Sigma-Aldrich) monoclonal antibody (diluted 1∶1000 in 0.5% (w/v) BSA in PBS). Cells were then washed three times with PBS and incubated for 1 h at 37°C with the fluorescein isothiocyanate (FITC)-conjugated anti-mouse IgG (Sigma-Aldrich) diluted 1∶100 and washed three times again. Cell slides were mounted in Vectashield (Vector Laboratories, Burlingame, CA, USA) and their immunofluorescence was observed and recorded on an Axiovert 40CFL fluorescence microscope (Carl Zeiss, Jena, Germany) equipped with an Axiocam MRc5 (Carl Zeiss) camera. Images were processed with the software AxioVision Rel. 4.6.3 (Carl Zeiss).

### 2DE

Bacterial total protein extracts and their separation by 2DE was performed as previously described [35]. Briefly, 800–1000 µg of protein in 450 µL re-hydration buffer were loaded onto 18 cm Immobiline DryStrips (GE Healthcare, Uppsala, Sweden) with a non-linear wide range pH gradient (pH 3–11). After active gel strip re-hydration, IEF was run on an Ettan IPGphor 3 unit (GE Healthcare) for a total of 100 kVh during which the voltage was gradually increased up to 5,000 V for a total of 66 h. For SDS-PAGE, the second dimension analysis, the proteins on the gel strips were equilibrated by soaking the strip in equilibration buffer for 15 min at RT and then for another 15 min in blocking buffer. Strips were finally applied onto 7–16% (w/v) gradient polyacrylamide gels and run overnight at 1 W/gel (Ettan DALT*six* System, GE Healthcare). Protein visualization was carried out by CBB staining (Sigma Aldrich).

### 2DE map analysis

CBB-stained 2DE gels were scanned in the ImageScanner (GE Healthcare) operated by the software LabScan 5 (GE Healthcare) in transparency mode with a red color filter. Scanning was carried out at 300 dpi and 16 bit grayscale. Images were analyzed using the ImageMaster™ 2D Platinum software (GE Healthcare) as before [35], taking into account the standardized relative intensity volume of spots (or % Vol, *i.e.* the volume of each spot over the volume of all spots in the gel). Differences in protein abundance among strains were statistically assessed. To assure result reproducibility, all samples were subjected to 2DE twice, making a total of 20 analyzed 2DE gels.

### Sample preparation and mass spectrometry analysis

The selected differentially expressed proteins were identified by PMF using an Autoflex III MALDI-TOF/TOF mass spectrometer (Bruker Daltonics, Bremen, Germany), as previously reported, with some minor modifications [35]. Protein spots were excised from the CBB-stained 2DE gels and enzymatically digested in-gel with proteomics grade porcine trypsin (Sigma-Aldrich). Trypsin-digested peptides were loaded in a disposable ready-to-use MALDI target prespotted with α-cyano-4-hydroxycinnaminic acid (Prespotted AnchorChip PAC 384/96, Bruker Daltonics) and were assayed with the mass spectrometer in the positive ion mode. Spectra acquired with the FlexControl (Compass software, version 1.2, Bruker Daltonics) in reflection mode, were processed by using FlexAnalysis (Compass software, version 1.2, Bruker Daltonics). Monoisotopic peptide masses were used to search protein databases (*H. pylori* NCBI nr. 2011.03.04, 13254464 sequences; or Swissprot databases nr. 2010.10, 521016 sequences) using MASCOT software (version 2.3.01, Matrix Science, London, UK). A mass accuracy of 50–100 ppm and 1 missed cleavage were allowed in the searches. In the absence of matches, the mass window was extended up to 200 ppm. Cysteine carbamidomethylation was considered as a fixed modification and protein *N*-terminal acetylation, oxidation of methionine and pyroglutamic-acid on N-terminal glutamic acid were allowed as variable modifications. Proteins with significant MOWSE scores (*p*<0.05) are reported. In a few special cases, MS identification based on MOWSE scores with no statistical significance were also reported and discussed since the same identification was obtained for the equivalent spot from different 2DE gels and because it matched the identification in the 2DE *H. pylori* database.

### Motility assays

5 µl of each pool of *H. pylori* prepared as mention above, were inoculated with a sterile pipette tip into motility agar plates, consisting of brain-heart infusion (BHI) medium (Oxoid, Hampshire, UK) supplemented with 5% (v/v) of heat-inactivated (56°C for 30 min) FBS, *H. pylori* selective supplement (10 mg/L of vancomycin, 5 mg/L of trimethoprim, 5 mg/L of cefsulodin and 5 mg/L of amphotericin B) (Oxoid) and 0.35% (w/v) agar. Motility was scored by measuring the bacterial halo diameter at 5, 7 and 11 days of incubation of the plates under microaerobic conditions (Campygen CN0025, Oxoid), at 37°C. Differences in motility of the two pools of strains, tested in three independent experiments, were statistically assessed.

### Statistics

Whenever necessary, differences were tested by Student's *t* test, being considered as statistically significant when *p*<0.05. Results are expressed as averages ± standard deviations (SD) of *n* observations.
